# Supplementary material for: Mitogenic and progenitor gene programmes in single pilocytic astrocytoma cells
Source: Nat Commun. 2019 Aug 19;10:3731. doi: 10.1038/s41467-019-11493-2 (PMC6700116; doi:10.1038/s41467-019-11493-2)
Supplement: Supplementary file 1 — Supplementary Information [file 41467_2019_11493_MOESM1_ESM.pdf]

Supplementary Information

Mitogenic and progenitor gene programs in single pilocytic astrocytoma cells

Reitman et al.

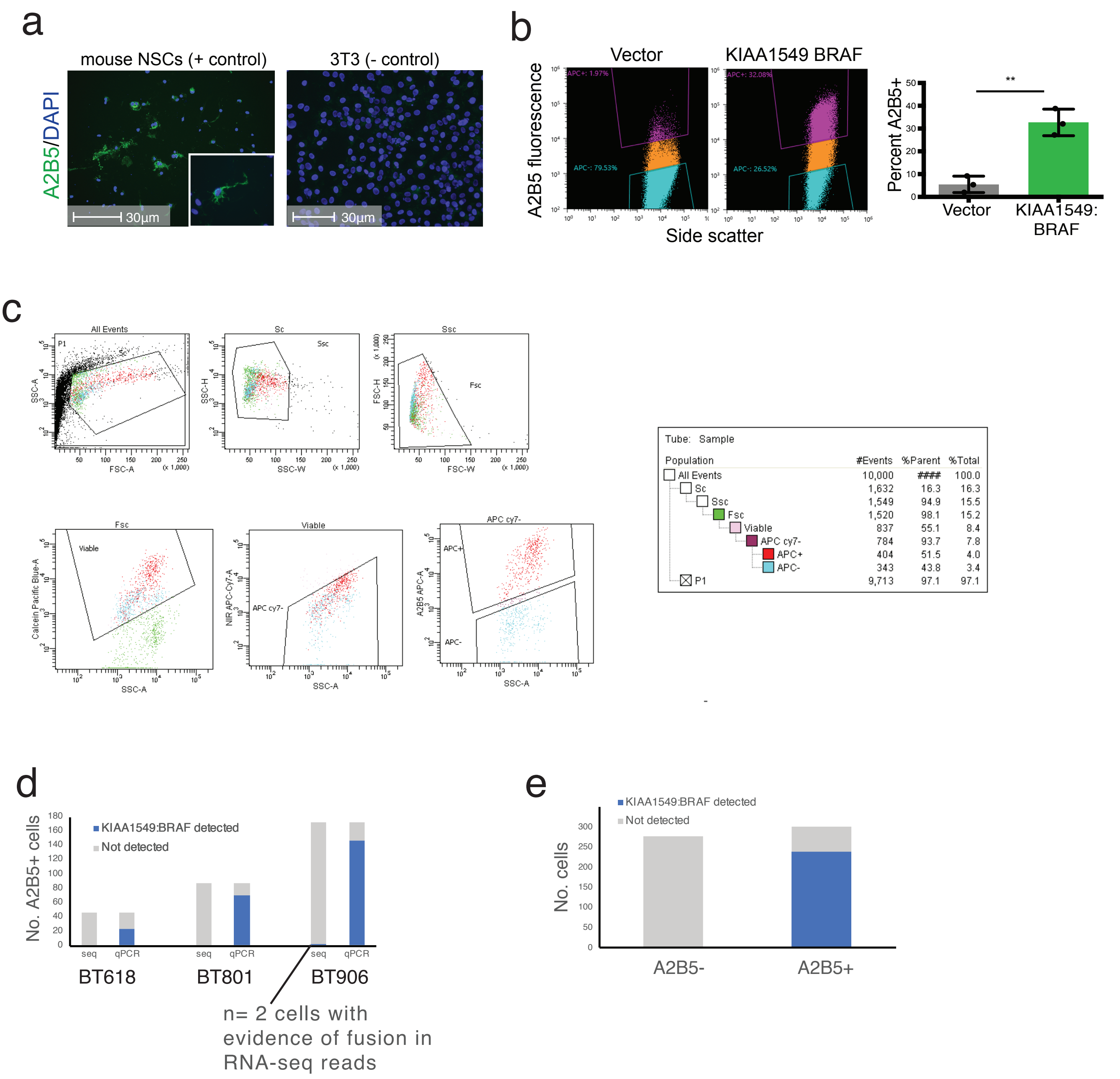

Supplementary Fig. 1: A2B5 staining and BRAF fusion detection in mouse neural stem cells and PA cells. **a,** A2B5 staining in mouse neural stem cells (mNSCs) and mouse fibroblast 3T3 cells. **b,** A2B5 status of mouse neural stem cells transfected with KIAA1549-BRAF or vector alone. Flow cytometry assessment of A2B5 fluorescence and quantification are shown. Data represent mean and error bars represent S.D. from n=3 experiments. **P = 0.0023, Student's t test. **c,** Representative flow cytometry plots showing gating strategy for one tumor (BT801). SSC, side scatter; FSC, forward scatter; NIR, near IR dead stain. **d,** Detection of KIAA1549-BRAF fusion in three PA tumors based on analysis of next generation sequencing reads (seq) or on quantitative PCR (qPCR). **e,** Number of A2B5 negative and A2B5 positive cells with KIAA1549-BRAF fusion detected by qPCR among three tumors.

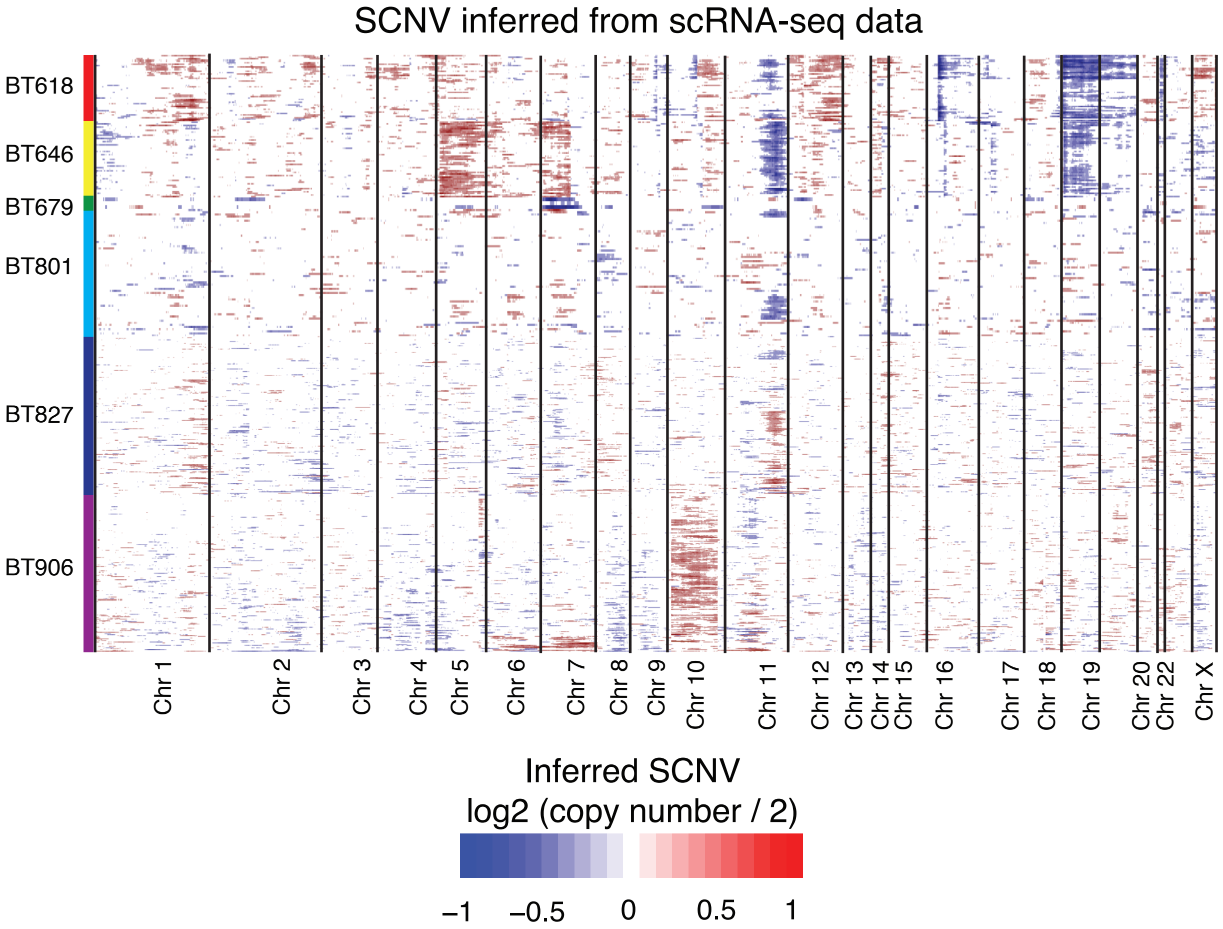

Supplementary Fig. 2: Inferral of somatic copy number variations (SCNVs) in PA cancer cells. Heat map showing SCNVs inferred by averaging gene expression over 100-gene windows and comparison to reference cells. We did not detect correlations between SCNVs and clinical outcomes of the patients.

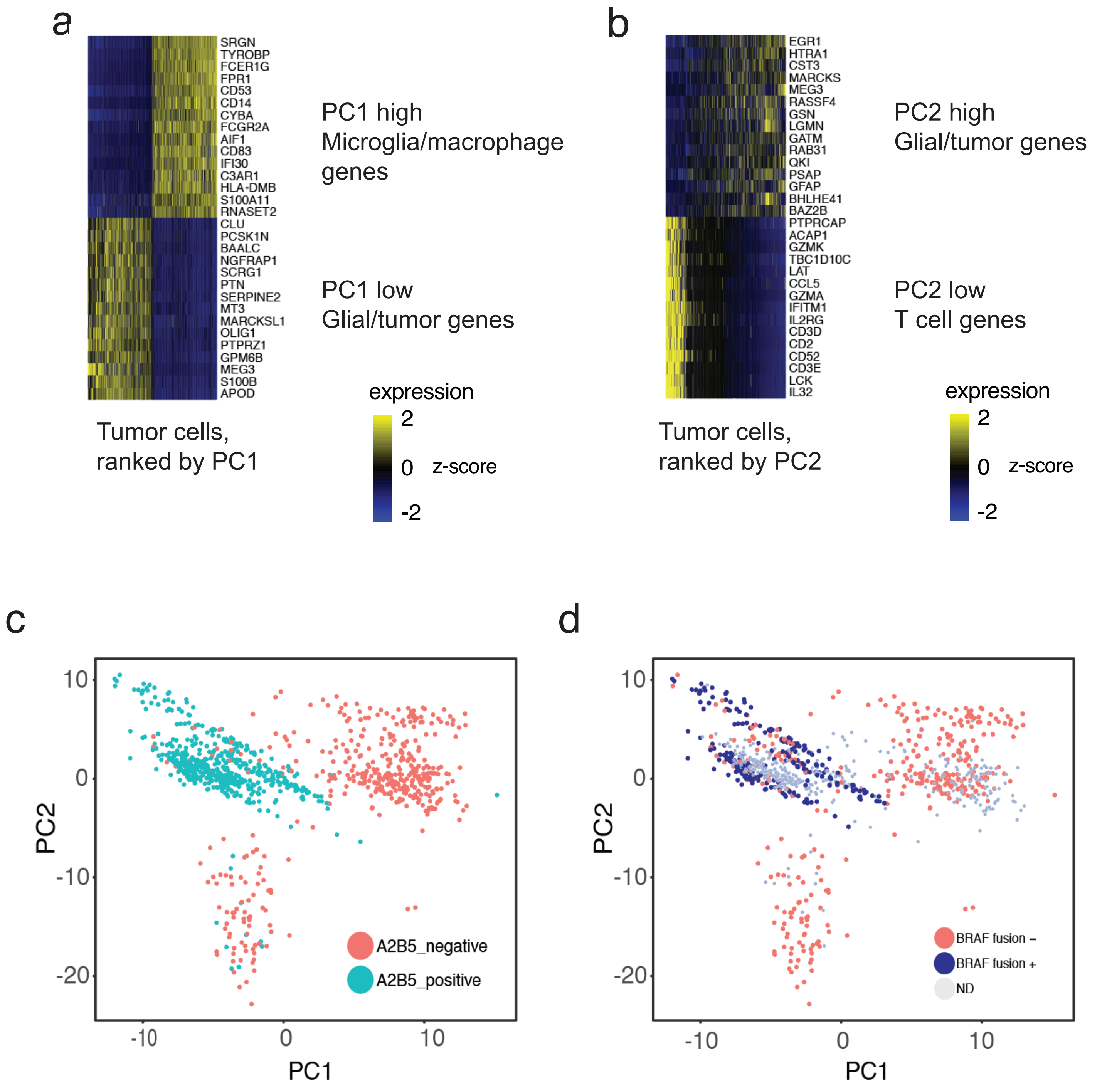

Supplementary Fig. 3: Principal component analysis of tumor and immune PA cells. a, Heat map showing top principal component 1 (PC1) genes representing immune genes, and bottom PC1 genes representing glial and tumor genes, among single PA cells. **b,** Top PC2 genes representing glial and tumor genes, and bottom PC1 genes representing T lymphocyte genes. **c,** Plot of PC1 vs. PC2 for all PA cells. Cells are colored by A2B5 status. **d,** Plot of PC1 vs. PC2 with cells colored by KIAA1549-BRAF fusion status as determined by BRAF Spike-in-Seq and BRAF fusion qPCR.

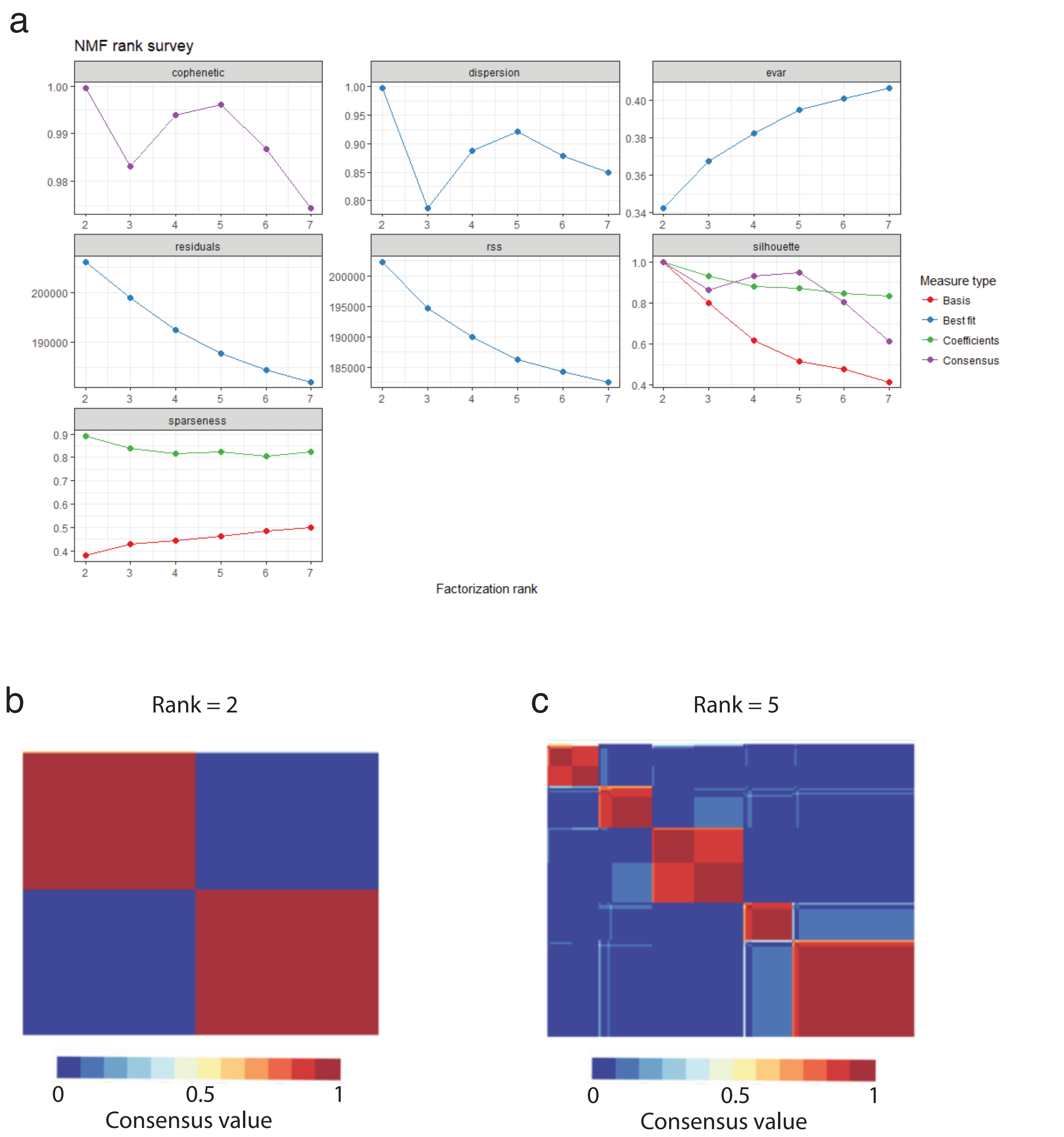

Supplementary Fig. 4: Non-negative matrix factorization (NMF) analysis of PA cells. a, Summary statistics for NMF analyses performed using two to seven ranks. The higher cophenetic coefficient values at ranks of 2 and 5 (top left panel) suggest greater stability when the dataset is divided into 2 or 5 clusters compared to different numbers of clusters. **b,** Heat map showing all NMF consensus values for all PA cells using rank = 2. **c,** Heat map showing all NMF consensus values for all PA cells using rank = 5.

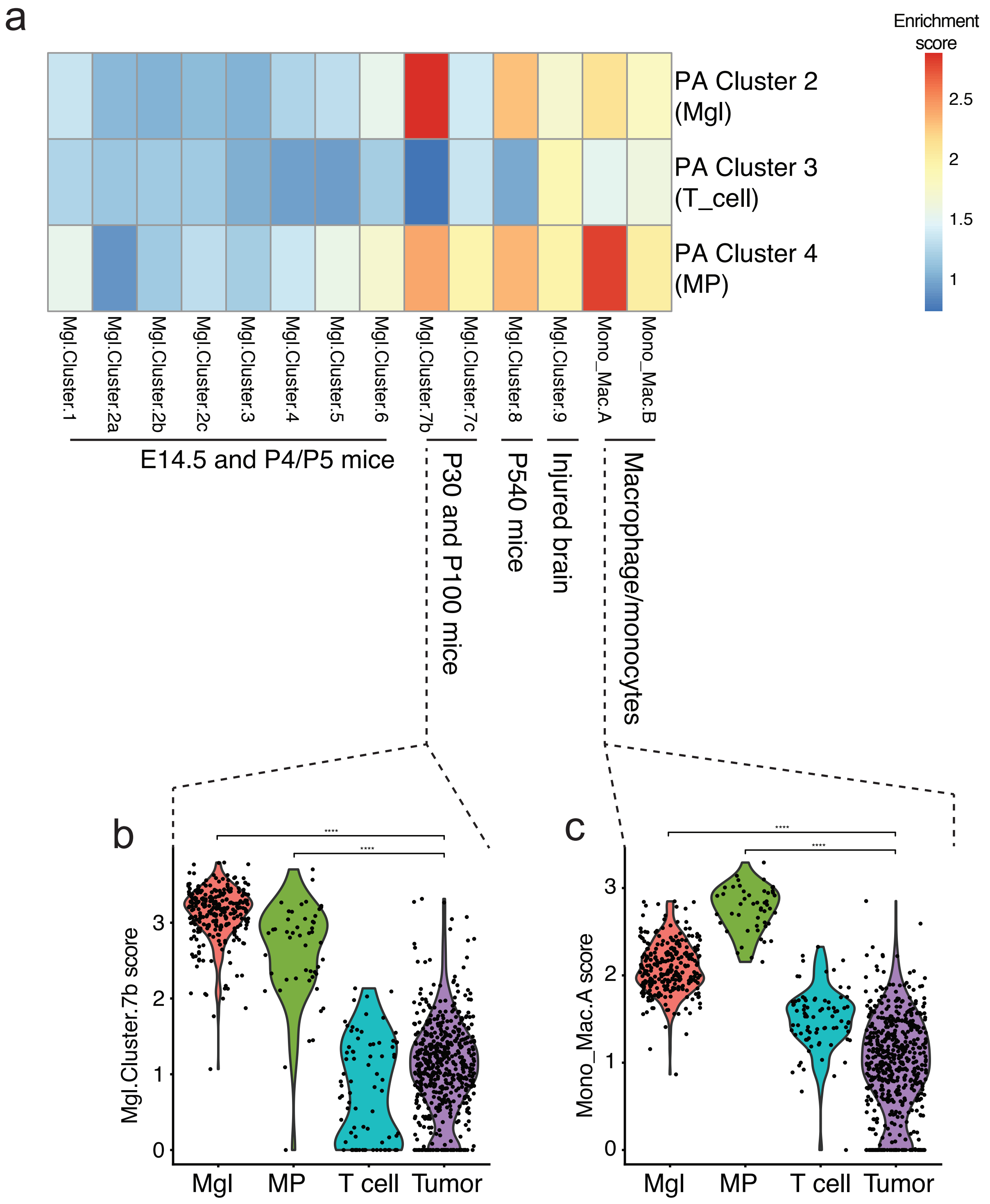

Supplementary Fig. 5: PA tumor-associated cells are enriched for specific microglia and macrophage gene signatures. a, Heat map showing mean enrichment scores for gene signatures of microglia derived from a single cell RNA-seq atlas of microglia collected throughout the mouse lifespan, in health and disease1 (x-axis) for PA cells in three immune cell clusters (y-axis). **b,** Violin plot showing enrichment scores for Cluster 7b microglia, which are a cluster of microglia derived from healthy adult mice[1]. **c,** Violin plot showing enrichment scores for Monocyte/Macrophage cluster A, which are a cluster of macrophages/monocytes derived from healthy adult mice[1]. Mgl, microglia; MP, macrophage. Pairwise comparisons of Mgl and MP clusters to reference tumor clusters are shown; ****P<0.0001, Wilcoxon rank sum test.

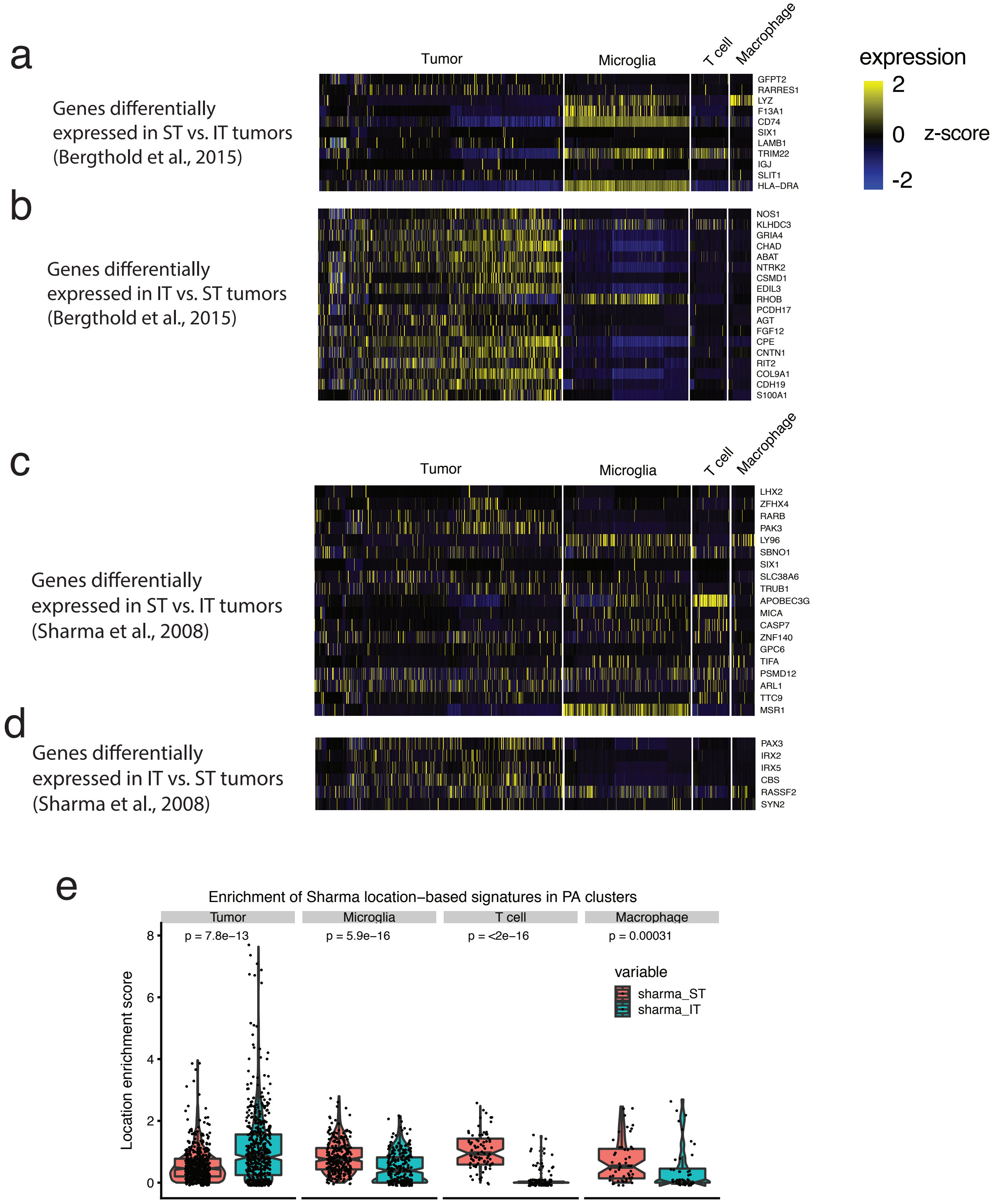

Supplementary Fig. 6: Expression of genes associated with brain location in PA cells. a, Heat map showing genes found to be differentially expressed in supratentorial (ST) vs. infratentorial (IT) pediatric low grade gliomas in our previous report (Bergthold and colleagues[2]). **b,** Heat map showing genes found to be differentially expressed in IT vs. ST pediatric low grade gliomas[2]. **c,** Heat map showing genes found to be differentially expressed in ST vs. IT pediatric low grade gliomas (Sharma and colleagues[3]). **d,** Heat map showing genes found to be differentially expressed in IT vs. ST pediatric low grade gliomas[3]. **e,** Plot showing enrichment of ST and IT gene signatures in PA cells from tumor, microglia, T cell, and macrophage clusters. Centre line shows median, hinges show 1st through 3rd quartiles, and whiskers extend to minimum and maximum data points.

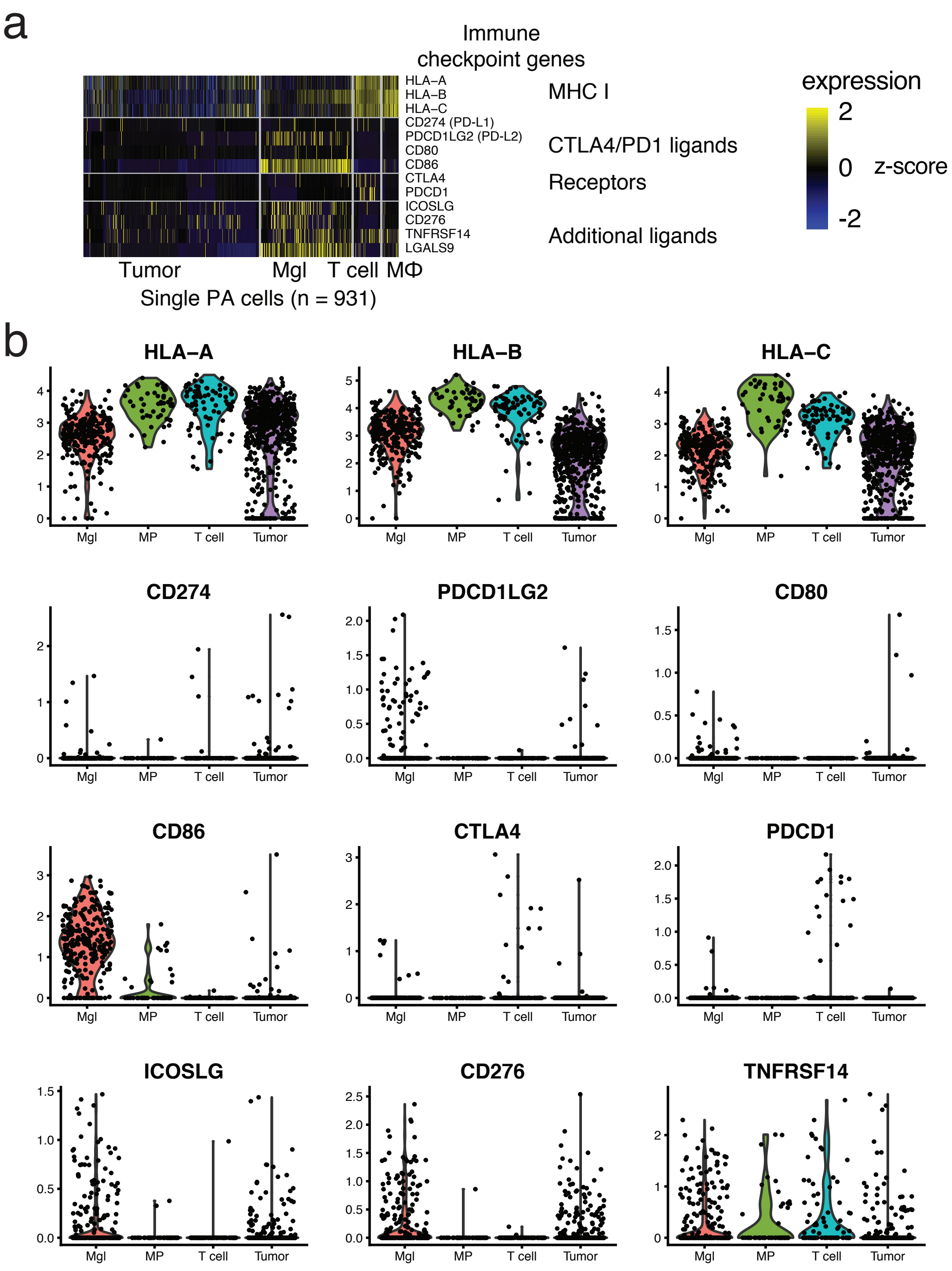

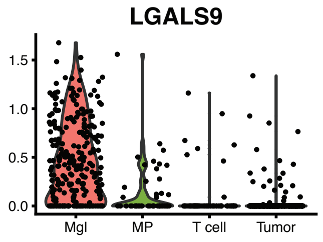

Supplementary Fig. 7: Immune checkpoint signaling analysis in tumor, antigen presenting cell, and T lymphocyte compartments. a, Heat map showing scaled expression of major histocompatibility complex genes, immune checkpoint receptor genes, and immune checkpoint ligand genes in single PA cells. Cells are arranged into tumor, microglia, T lymphocyte, and macrophage (MΦ) groups. **b,** Violin plots showing log-normalized read counts for major histocompatibility complex genes, immune checkpoint receptor genes, and immune checkpoint ligand genes in single PA cells. Cells are arranged into tumor, microglia, T lymphocyte, and macrophage (MΦ) groups.

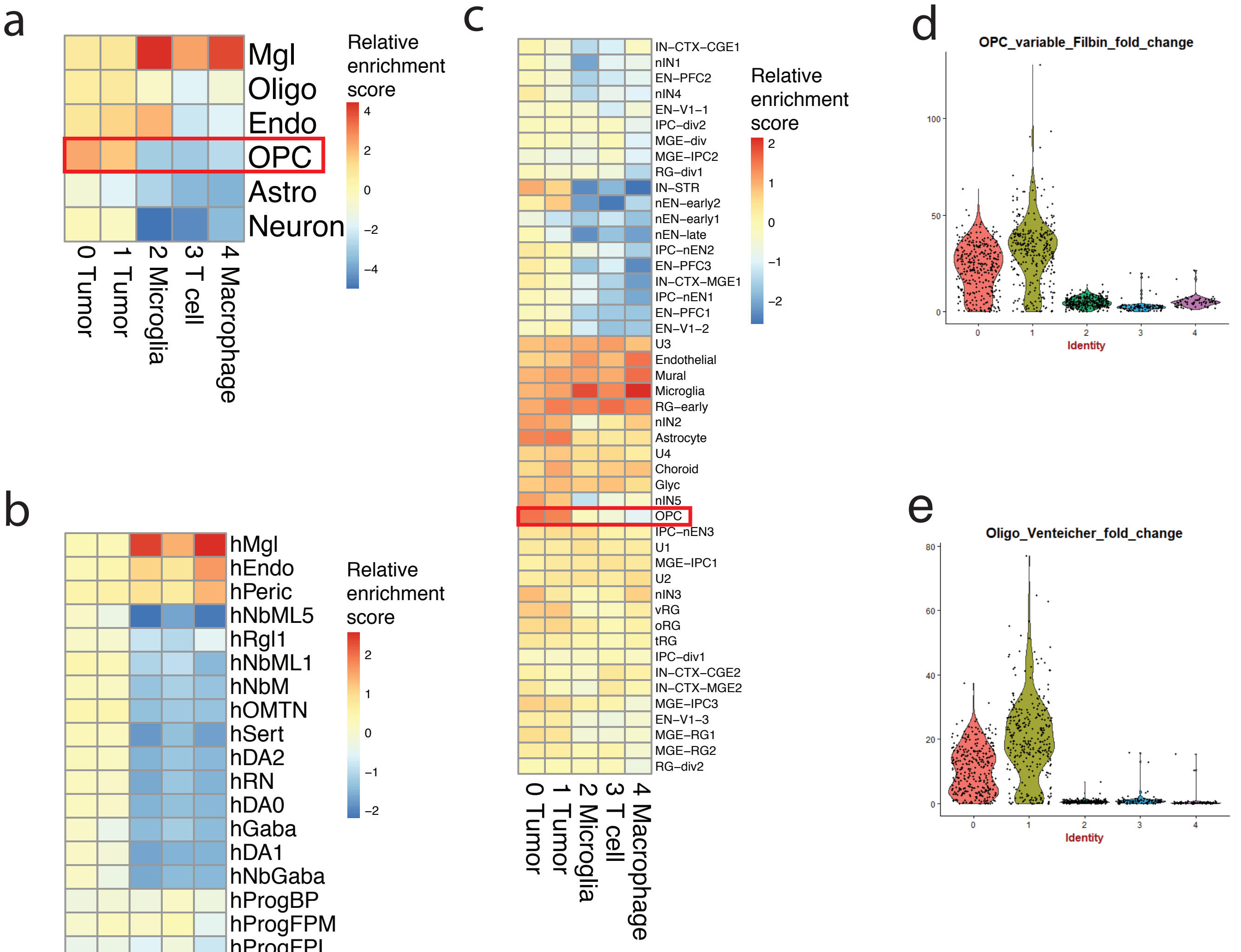

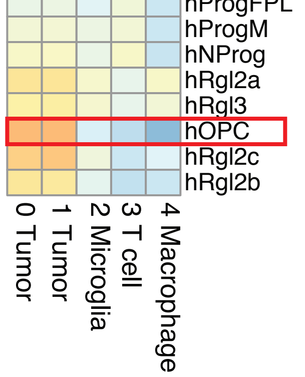

Supplementary Fig. 8: Comparison of tumor and immune cell clusters to normal brain and high-grade tumor gene signatures. a, Heat map showing mean enrichment of gene signatures associated with normal human brain cell types (rows) in PA tumor and immune cell clusters (columns). Brain cell types are from a human adult cortex single cell RNA-seq atlas[4]. **b,** Heat map showing enrichment of gene signatures associated with human developing midbrain cells[5] (rows) in PA cell clusters (columns). **c,** Heat map showing enrichment of gene signatures associated with human developing cortex cells[6] (rows) in PA cell clusters (columns). **d,** Violin plot showing enrichment of OPC_variable gene program seen in pediatric high-grade midline gliomas[7] in PA cells, arranged by cluster. **e,** Violin plot showing enrichment of oligodendrocyte-like gene program seen in IDH-mutated intermediate-grade gliomas[8] in PA cells, arranged by cluster.

Abbreviations: OPC, oligodendrocyte precursor cell (boxed in red for emphasis). Oligo, oligodendrocyte; Astro, astrocyte; Mgl, microglia; Endo, endothelial cell; OMTN, oculomotor and trochlear nucleus; Sert, serotonergic; NbM, medial neuroblast; NbDA, neuroblast dopaminergic; DA0-2, dopaminergic neurons; RN, red nucleus; Gaba1-2, GABAergic neurons; mNbL1-2, lateral neuroblasts; NbML1-5, mediolateral neuroblasts; NProg, neuronal progenitor; Prog, progenitor medial floorplate (FPM), lateral floorplate (FPL), midline (M), basal plate (BP); Rgl1-3, radial glia-like cells; Mgl, microglia; Endo, endothelial cells; Peric, pericytes; Epend, ependymal; IN-CTX-CGE, central ganglionic eminence-derived inhibitory neurons; IPC-div1/2, dividing intermediate radial glia-like progenitor cells; MGE-div, dividing MGE progenitors; RG-div1, dividing radial glia G2/M-phase; RG-div2, dividing radial glia (S-phase); EN-PFC, early and late born excitatory neuron prefrontal cortex; EN-V1-2, early born deep layer excitatory neuron V1-2; RG-early, early radial glia; Glyc, glycolysis; IPC-nEN1/2/3, non-dividing intermediate progenitor cells; nIN1/2/3/4/5, medial ganglionic eminence neurons; MGE-IPC1/2/3, medial ganglionic eminence progenitors; MGE-RG1/2, MGE radial glia; IN-CTX-MGE1/2, MGE-derived inhibitory neuron; nEN-early1/2, newborn excitatory neuron; nEN-late, late-born excitatory neuron; OPC, oligodendrocyte precursor cell; oRG, outer radial glia; IN-STR, striatal neurons; tRG, truncated radial glia; U1/2/3/4, unknown cell type; vRG, ventricular radial glia.

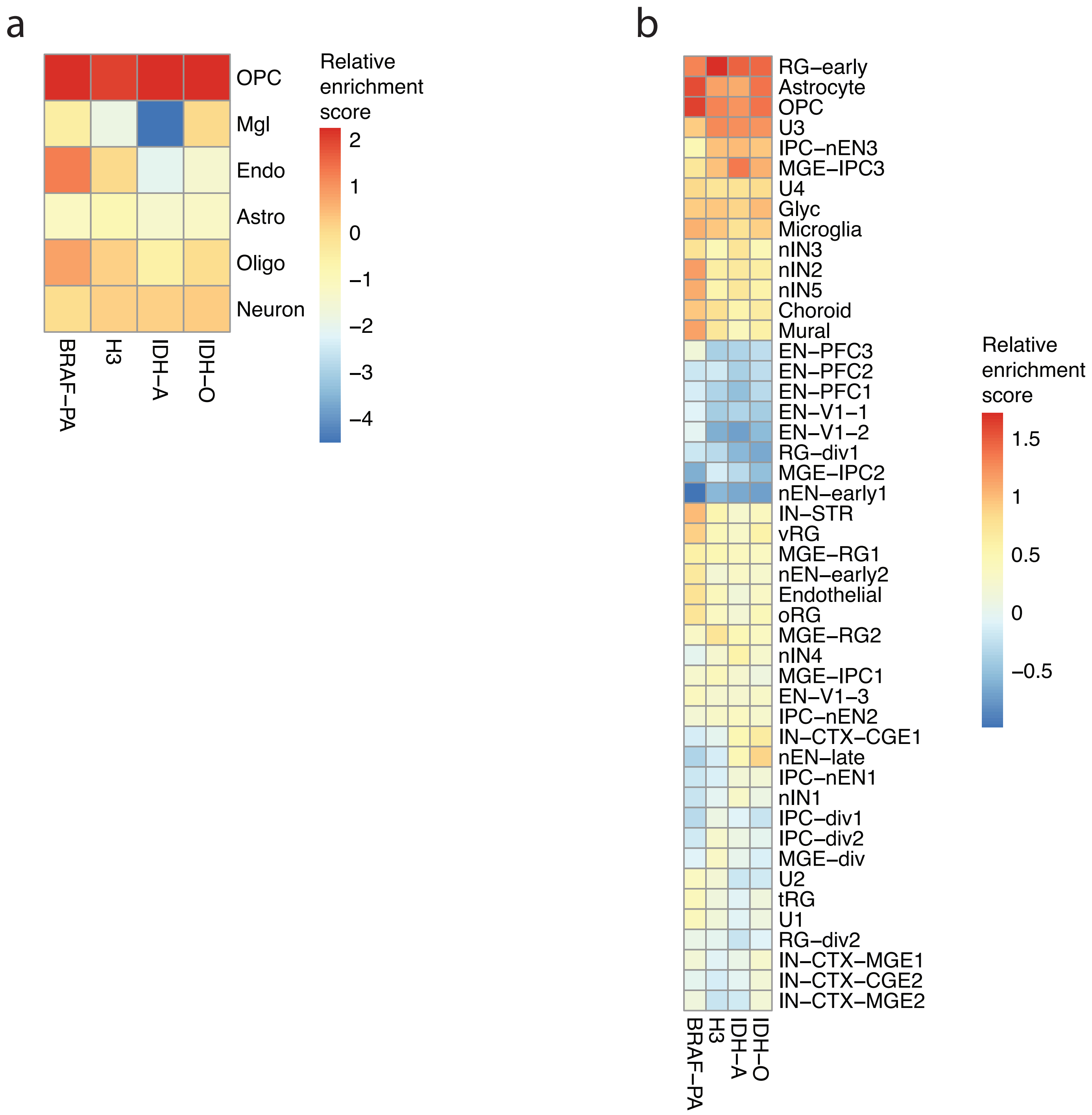

Supplementary Fig. 9: Comparison of PA and higher-grade gliomas to normal brain gene signatures. a, Heat map showing mean enrichment of gene signatures associated with normal human brain cell types (rows) in different glioma tumor types (columns). Tumor types include PA (BRAF-PA), IDH-mutated oligodendrogliomas[9] (IDH-O), IDH-mutated astrocytoma[8] (IDH-A), and H3K27M-mutated pediatric midline glioma[7] (H3). Brain cell types are from a human adult cortex single cell RNA-seq atlas[4]. **b,** Heat map showing enrichment of gene signatures associated with human developing cortex cells (rows) in PA compared to higher grade gliomas (columns). Cell type abbreviations are the same as for Supplementary Figure 8.

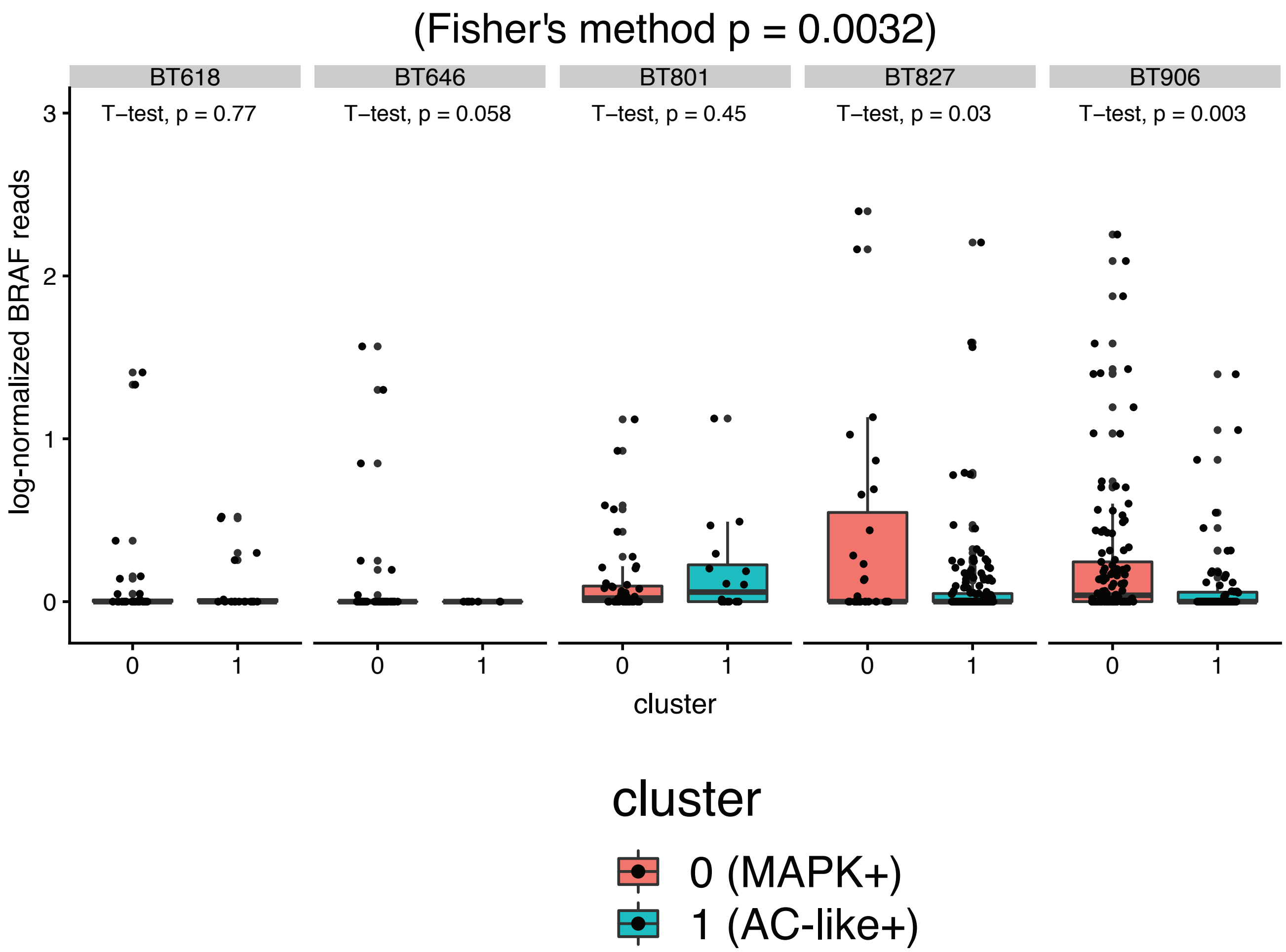

Supplementary Fig. 10: BRAF expression in MAPK and AC-like PA cancer cells. Expression of BRAF-derived RNA-seq reads in cells that predominantly express the MAPK gene program (cluster 0) and PA cancer cells that predominantly express the AC-like gene program (cluster 1) are shown for cancer cells from for five PA tumors. Student's t-test P-values are shown to compare differences in mean between groups within each tumor, and an aggregated P-value for all samples using Fisher's method is shown. Centre line shows median, hinges show 1st through 3rd quartiles, and whiskers extend from boxes to 1.5 times the adjacent interquartile range.

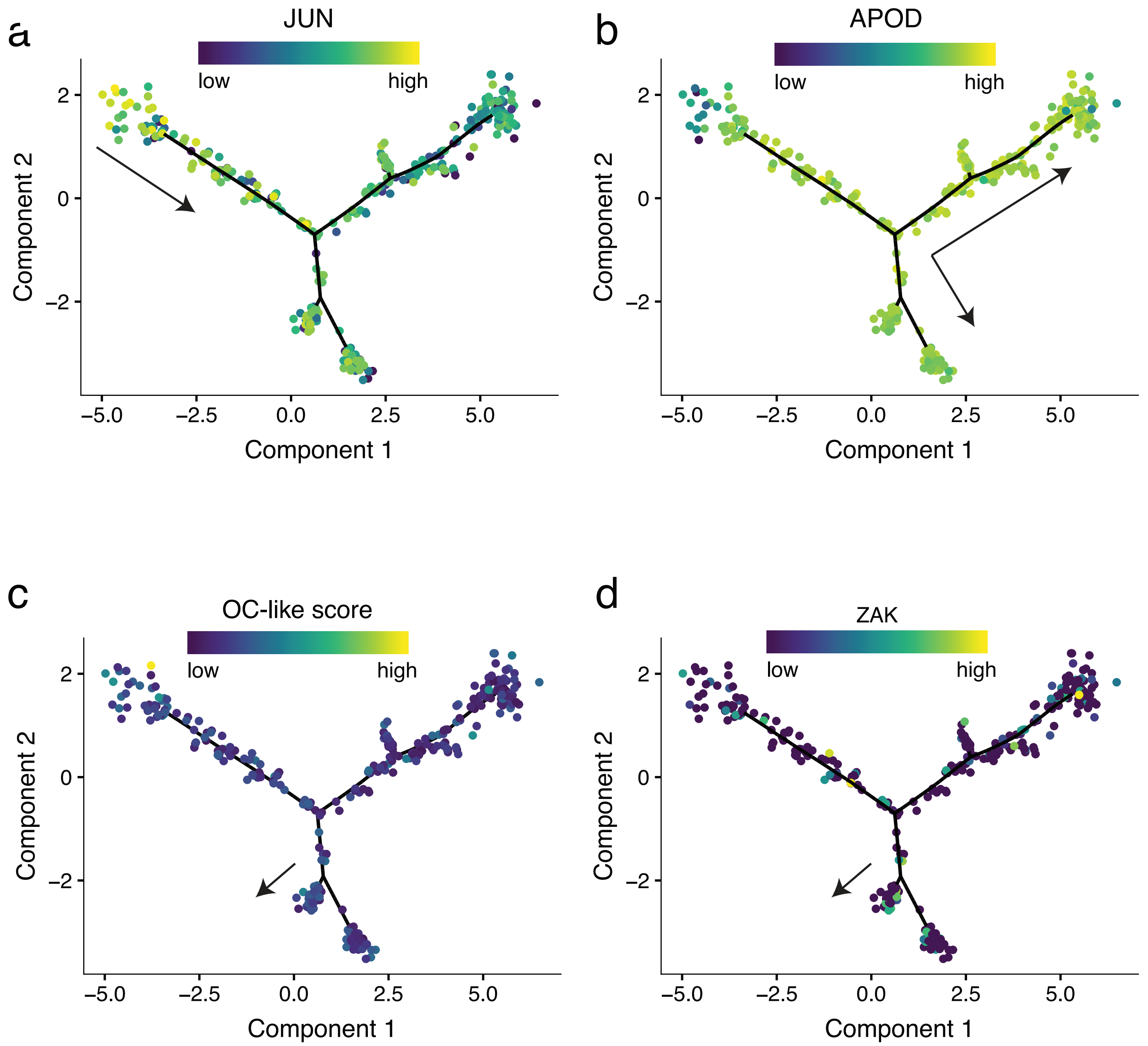

Supplementary Fig. 11: Gene program markers in an inferred PA developmental trajectory. PA cancer cells are plotted based on a lineage trajectory inferred from RNA-seq data. **a,** Expression of JUN, a top marker gene for the MAPK signaling gene program. **b,** Expression of APOD, a top marker of the AC-like gene program; **c,** OC-like gene program score. **d,** Expression of ZAK, a top marker gene for the OC-like gene program. Gene expression is based on log-normalized transcripts per million.

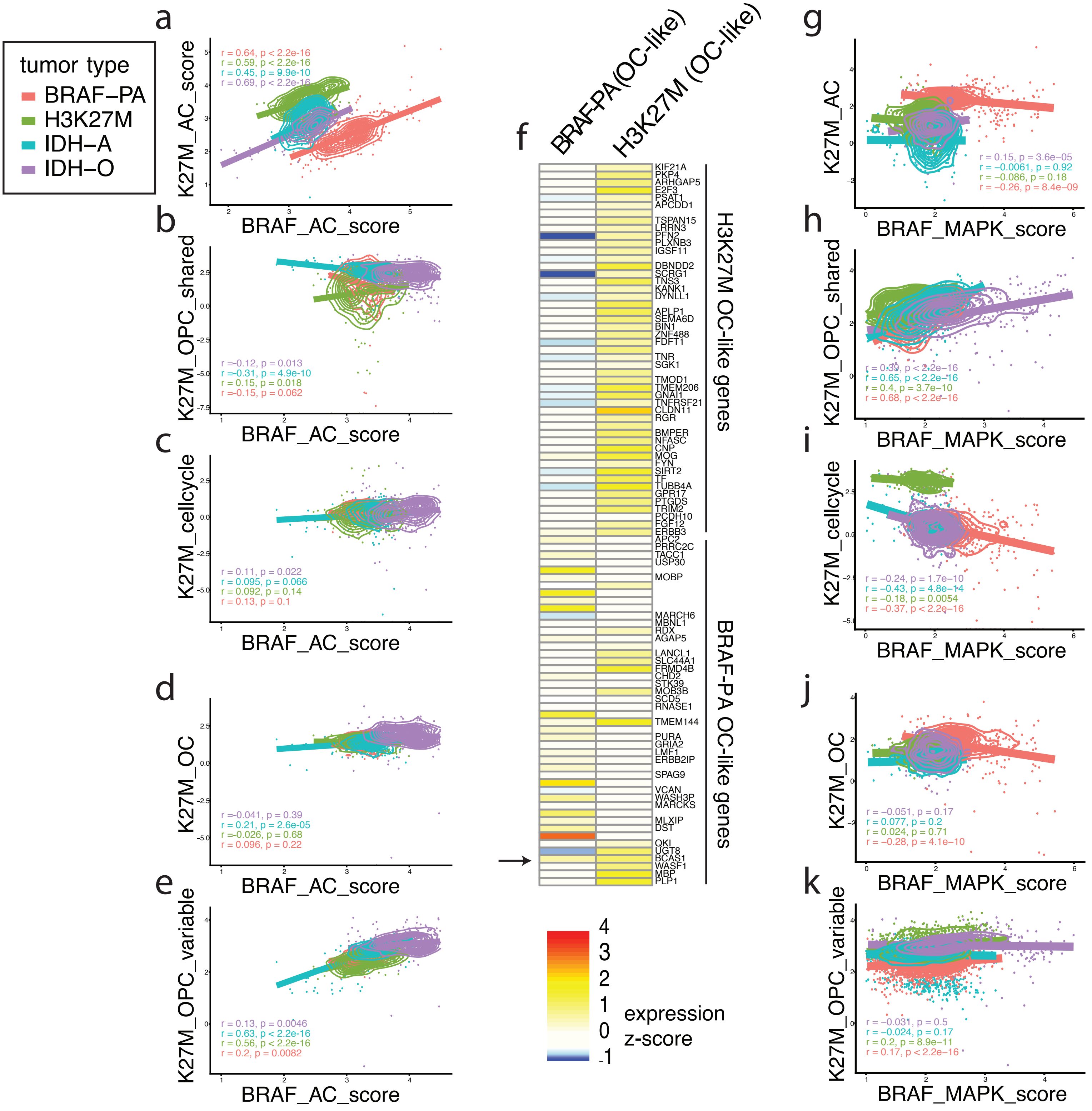

Supplementary Fig. 12: Gene program comparisons between PA and higher-grade gliomas. a-e, Plots showing AC-like cancer cells from PA (BRAF-PA), H3K27M midline gliomas, IDH-mutated astrocytomas (IDH-A), and IDH-mutated oligodendrogliomas (IDH-O). Cells are plotted by PA-derived AC-like gene program enrichment score (x-axis) and enrichment scores for AC-like (**a**), OPC-shared (**b**), stem/cell-cycle (**c**), OC-like (**d**), and OPC-variable (**e**) gene programs derived from high-grade H3K27M midline gliomas[7] (y-axes). Linear regression trendlines and Spearman correlation statistics are shown. **f,** Average expression of genes in OC-like signatures derived from PA and from H3K27M midline gliomas among OC-like cells from either tumor type. **g-k,** Plots showing PA cells expressing MAPK signaling program, H3K27M cells expressing OPC-like program, and IDH-A and IDH-O cells expressing a stem gene program. Cells are plotted by PA-derived MAPK signaling gene program enrichment score (x-axis) and enrichment scores for AC-like (**g**), OPC-shared (**h**), stem/cell-cycle (**i**), OC-like (**j**), and OPC-variable (**k**) gene programs derived from high-grade H3K27M midline gliomas (y-axes). Linear regression trendlines and Spearman correlation statistics are shown.

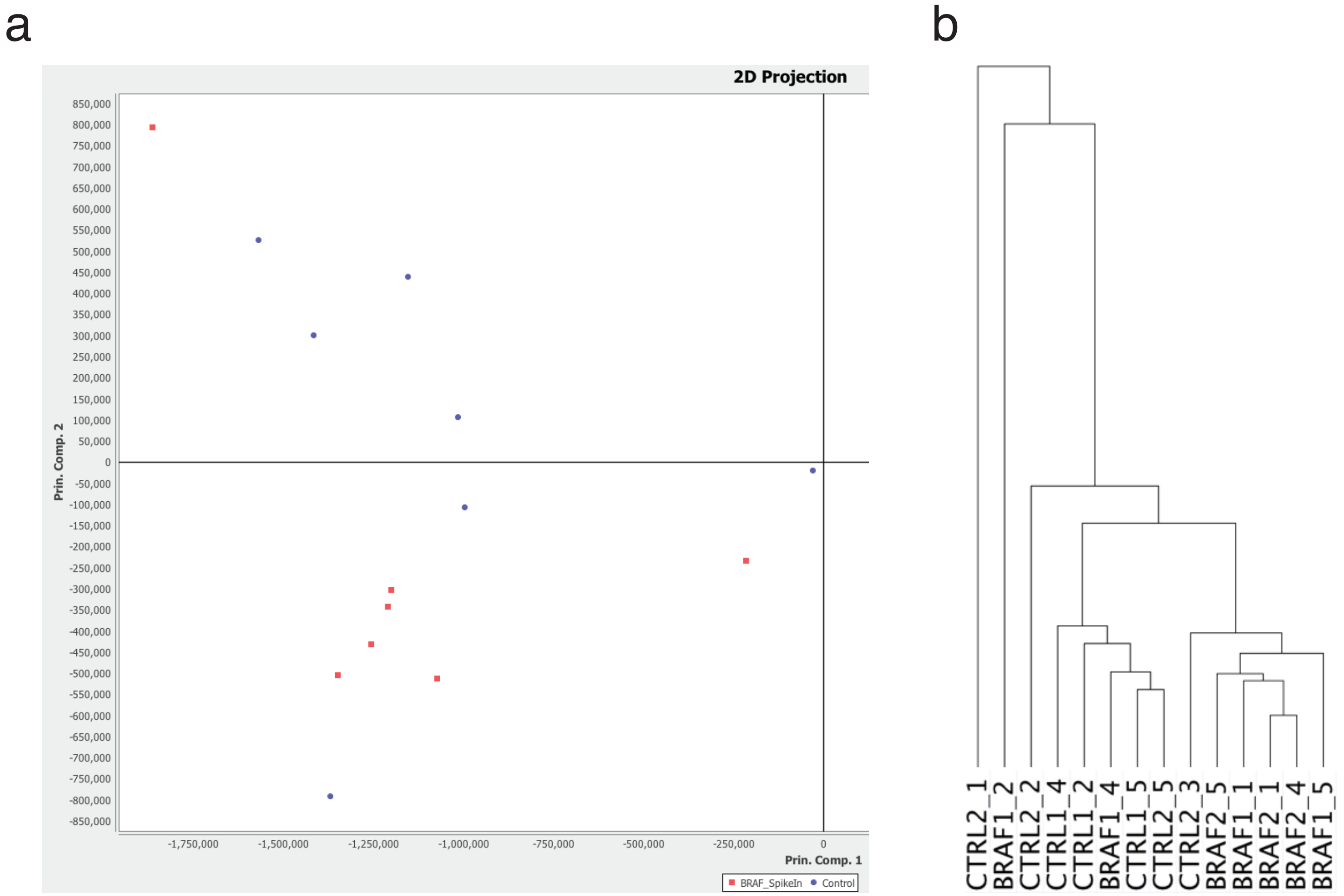

Supplementary Fig. 13: Comparison of scRNA-seq workflows with and without BRAF oligonucleotide spike-in. a, Plot showing top two principal components for mouse neural stem cell scRNAseq data derived from cells processed with BRAF oligonucleotide spike-in workflow (n=6) and without BRAF oligonucleotide spike-in (n=6). **b,** Hierarchical clustering of the same mouse neural stem cell scRNAseq data for cells processed with BRAF oligonucleotide spike-in workflow (BRAF, n=6) and without BRAF oligonucleotide spike-in (CTRL, n=6).

Supplementary References

1. Hammond, T.R. *et al.* Single-Cell RNA Sequencing of Microglia throughout the Mouse Lifespan and in the Injured Brain Reveals Complex Cell-State Changes. *Immunity* (2018).
2. Bergthold, G. *et al.* Expression profiles of 151 pediatric low-grade gliomas reveal molecular differences associated with location and histological subtype. *Neuro Oncol* **17**, 1486-96 (2015).
3. Sharma, M.K. *et al.* Distinct genetic signatures among pilocytic astrocytomas relate to their brain region origin. *Cancer Res* **67**, 890-900 (2007).
4. Darmanis, S. *et al.* A survey of human brain transcriptome diversity at the single cell level. *Proc Natl Acad Sci U S A* **112**, 7285-90 (2015).
5. La Manno, G. *et al.* Molecular Diversity of Midbrain Development in Mouse, Human, and Stem Cells. *Cell* **167**, 566-580 e19 (2016).
6. Nowakowski, T.J. *et al.* Spatiotemporal gene expression trajectories reveal developmental hierarchies of the human cortex. *Science* **358**, 1318-1323 (2017).
7. Filbin, M.G. *et al.* Developmental and oncogenic programs in H3K27M gliomas dissected by single-cell RNA-seq. *Science* **360**, 331-335 (2018).
8. Venteicher, A.S. *et al.* Decoupling genetics, lineages, and microenvironment in IDH-mutant gliomas by single-cell RNA-seq. *Science* **355**(2017).
9. Tirosh, I. *et al.* Single-cell RNA-seq supports a developmental hierarchy in human oligodendroglioma. *Nature* **539**, 309-313 (2016).
