# Supplementary material for: Mitogenic and progenitor gene programmes in single pilocytic astrocytoma cells
Source: Nat Commun. 2019 Aug 19;10:3731. doi: 10.1038/s41467-019-11493-2 (PMC6700116; doi:10.1038/s41467-019-11493-2)
Supplement: Supplementary file 3 — Description of Additional Supplementary Files [file 41467_2019_11493_MOESM3_ESM.pdf]

Description of Additional Supplementary Files

File Name: Supplementary Data 1
Description: Patient characteristics

File Name: Supplementary Data 2
Description: Genetic alterations in tumor bulk tissues

File Name: Supplementary Data 3
Description: Immune cluster differentially expressed genes

File Name: Supplementary Data 4
Description: Cancer cell differentially expressed genes

File Name: Supplementary Data 5
Description: Gene set enrichment analysis based on cancer cell differentially expressed genes

File Name: Supplementary Data 6
Description: Cancer cell gene programs

File Name: Supplementary Data 7
Description: Sequences of oligonucleotides used in this study
