# Supplementary material for: Mitogenic and progenitor gene programmes in single pilocytic astrocytoma cells
Source: Nat Commun. 2019 Aug 19;10:3731. doi: 10.1038/s41467-019-11493-2 (PMC6700116; doi:10.1038/s41467-019-11493-2)
Supplement: Supplementary file 11 — Reporting Summary [file 41467_2019_11493_MOESM11_ESM.pdf]

nature research

Corresponding author(s): Pratiti Bandopadhayay, Rameen Beroukhim

Reporting Summary

Nature Research wishes to improve the reproducibility of the work that we publish. This form provides structure for consistency and transparency in reporting. For further information on Nature Research policies, see Authors & Referees and the Editorial Policy Checklist.

Statistical parameters

When statistical analyses are reported, confirm that the following items are present in the relevant location (e.g. figure legend, table legend, main text, or Methods section).

n/a	Confirmed	
☐	☒	The exact sample size (*n*) for each experimental group/condition, given as a discrete number and unit of measurement
☐	☒	An indication of whether measurements were taken from distinct samples or whether the same sample was measured repeatedly
☐	☒	The statistical test(s) used AND whether they are one- or two-sided<br>*Only common tests should be described solely by name; describe more complex techniques in the Methods section.*
☒	☐	A description of all covariates tested
☐	☒	A description of any assumptions or corrections, such as tests of normality and adjustment for multiple comparisons
☒	☐	A full description of the statistics including central tendency (e.g. means) or other basic estimates (e.g. regression coefficient) AND variation (e.g. standard deviation) or associated estimates of uncertainty (e.g. confidence intervals)
☐	☒	For null hypothesis testing, the test statistic (e.g. *F*, *t*, *r*) with confidence intervals, effect sizes, degrees of freedom and *P* value noted<br>*Give P values as exact values whenever suitable.*
☒	☐	For Bayesian analysis, information on the choice of priors and Markov chain Monte Carlo settings
☒	☐	For hierarchical and complex designs, identification of the appropriate level for tests and full reporting of outcomes
☐	☒	Estimates of effect sizes (e.g. Cohen's *d*, Pearson's *r*), indicating how they were calculated
☐	☒	Clearly defined error bars<br>*State explicitly what error bars represent (e.g. SD, SE, CI)*

Our web collection on statistics for biologists may be useful.

Software and code

Policy information about availability of computer code

Data collection	Sequenced reads were aligned to the UCSC hg19 reference genome assembly using STAR v2.5.1b39 and gene expression was quantified using RSEM v1.2.2840 as part of the VIPER snakemake pipeline. KIAA1549-BRAF gene fusions were identified in the RNA-seq data using STAR-Fusion v0.5.4.42.
Data analysis	All processed data was analyzed in R v3.4.3. NMF was performed using R package NNMF. Transcripts per million data were log normalized and scaled using R package Seurat. PCA and shared nearest neighbor clustering was performed in R package Seurat. Visualizations were performed using Seurat43, ggplot2, and pheatmap.

For manuscripts utilizing custom algorithms or software that are central to the research but not yet described in published literature, software must be made available to editors/reviewers upon request. We strongly encourage code deposition in a community repository (e.g. GitHub). See the Nature Research guidelines for submitting code & software for further information.

Data

Policy information about availability of data

All manuscripts must include a data availability statement. This statement should provide the following information, where applicable:

- Accession codes, unique identifiers, or web links for publicly available datasets
- A list of figures that have associated raw data
- A description of any restrictions on data availability

Data availability. The human RNA sequencing data has been deposited in the dbGaP repository under the accession number phs001854.v1.p1 [https://www.ncbi.nlm.nih.gov/projects/gap/cgi-bin/study.cgi?study_id=phs001854.v1.p1]. Expression data and metadata for tumor of origin, cluster, and t-SNE coordinates for single cells have been uploaded to the Broad Institute Single Cell Portal under accession number SCP271 [https://portals.broadinstitute.org/single_cell/study/SCP271]. The mouse neural stem cell RNA sequencing data, expression matrix, and metadata has been deposited to the Single Cell Portal under the accession number SCP468 [https://portals.broadinstitute.org/single_cell/study/SCP468]. The source data underlying Figs 6a-j are provided as a Source Data file. The datasets for H3K27M mutant pediatric midline gliomas4, oligodendrogliomas2, and intermediate grade astrocytomas3 referenced in the study are available from the Single Cell Portal with accession numbers SCP147 [https://portals.broadinstitute.org/single_cell/study/SCP147], SCP12 [https://portals.broadinstitute.org/single_cell/study/SCP12], and SCP50 [https://portals.broadinstitute.org/single_cell/study/SCP50] respectively. Normal adult cortex gene signatures were derived from marker gene lists found in Figure 1 of a scRNA-seq atlas of normal adult cortex32. Developing midbrain gene signatures were derived from the marker gene matrix found in Table S2 of a publication by La Manno and colleagues31. Each midbrain cell type gene signature comprised the list of all genes expressed in that cell type in the marker gene matrix. Developing cortex gene signatures were derived from the differential gene list found in Table S5 of a publication by Nowakowski and colleagues33. For the developing cortex gene sets, only the differential genes with $P < 0.0005$ (non-parametric Wilcoxon rank sum test) were used for the gene signature for each cell type in order to restrict gene signatures to ~200 genes. All the other data supporting the findings of this paper are available within the article, the supplementary information files and from the corresponding author upon reasonable request.

Field-specific reporting

Please select the best fit for your research. If you are not sure, read the appropriate sections before making your selection.

☒ Life sciences ☐ Behavioural & social sciences ☐ Ecological, evolutionary & environmental sciences

For a reference copy of the document with all sections, see nature.com/authors/policies/ReportingSummary-flat.pdf

Life sciences study design

All studies must disclose on these points even when the disclosure is negative.

Sample size	n = 6 tumors were studied.
Data exclusions	No data were excluded.
Replication	Replicates were performed to confirm reproducibility of all in vitro mouse neural stem cell experiments.
Randomization	This is not relevant to the current study because no randomization or treatment selection was necessary
Blinding	Not relevant because a sample response based on treatment/conditions was not done

Reporting for specific materials, systems and methods

Materials & experimental systems

n/a	Involved in the study	
☒	☐	Unique biological materials
☐	☒	Antibodies
☐	☒	Eukaryotic cell lines
☒	☐	Palaeontology
☒	☐	Animals and other organisms
☐	☒	Human research participants

Methods

n/a	Involved in the study	
☒	☐	ChIP-seq
☐	☒	Flow cytometry
☒	☐	MRI-based neuroimaging

Antibodies

Antibodies used: A2B5 clone: 105HB29, Miltenyi Biotec. F-IHC antibody panel consisted of Olig2 (R&D System, Goat polyclonal, AF2418) with

	CY5.5, GFAP (Cell Signaling Techology, Mouse monoclonal, Clone GA5, #3670) with FITC, and Ki-67 (Dako, Mouse monoclonal, Clone MIB-1, M7240) with CY3.
Validation	A2B5 validated on mouse neural stem cells and control non-neuronal cells in Extended Data Figure 1.

Eukaryotic cell lines

Policy information about cell lines

Cell line source(s)	Mouse neuronal stem cells from Stiles lab as descrived in Sun et al., 2017, Neuro Oncol 19, 774-785
Authentication	Routine fingerprinting and mycoplasma testing was performed.
Mycoplasma contamination	All cell lines were routinely tested for mycoplasma and found to be negative.
Commonly misidentified lines (See ICLAC register)	None.

Human research participants

Policy information about studies involving human research participants

Population characteristics	Brain tumor solid tissues from patients undergoing tumor resections at Boston Children's Hospital from 2012-2016.
Recruitment	All pediatric patients undergoing brain tumor surgery were approached for consent to provide biospecimens.

Flow Cytometry

Plots

Confirm that:

☒ The axis labels state the marker and fluorochrome used (e.g. CD4-FITC).

☒ The axis scales are clearly visible. Include numbers along axes only for bottom left plot of group (a 'group' is an analysis of identical markers).

☒ All plots are contour plots with outliers or pseudocolor plots.

☒ A numerical value for number of cells or percentage (with statistics) is provided.

Methodology

Sample preparation	Tumor cells were pelleted and then resuspended in 1 ml 1x RBC lysis buffer (ThermoFisher) and incubated for 10 minutes at room temperature. Cells were washed with 10 mL 1x PBS then resuspended into 100 uL of FACS buffer (1x PBS with 1% BSA), counted, and then diluted to 1-5 million total cells/mL including non-viable cells. Cells were labeled with 10 uL anti-A2B5-APC (clone: 105HB29, Miltenyi Biotec) and 1.5 uL Calcein Blue AM (Invitrogen, cat# C1429) per 100 uL of cell suspension. Cells were pelleted, washed with 1 mL FACS buffer, and then resuspended into 500 uL FACS buffer containing 0.5 ul of a LIVE/DEAD fixable near IR dead cell stain kit (cat# L-34974).
Instrument	Sony SH800 flow sorter
Software	SH800 software
Cell population abundance	Approximately 30% of cells were determined for each.
Gating strategy	Calcein+, near IR dead-, A2B5+ cells or A2B5- cells were collected. Top 50% and bottom 50% of A2B5-staining cells were collected for each gate.

☒ Tick this box to confirm that a figure exemplifying the gating strategy is provided in the Supplementary Information.
